# Overexpression of Terpenoid Biosynthesis Genes From Garden Sage (*Salvia officinalis*) Modulates Rhizobia Interaction and Nodulation in Soybean

**DOI:** 10.3389/fpls.2021.783269

**Published:** 2021-12-23

**Authors:** Mohammed Ali, Long Miao, Qiuqiang Hou, Doaa B. Darwish, Salma Saleh Alrdahe, Ahmed Ali, Vagner A. Benedito, Million Tadege, Xiaobo Wang, Jian Zhao

**Affiliations:** ^1^Egyptian Deserts Gene Bank, North Sinai Research Station, Department of Genetic Resources, Desert Research Center, Cairo, Egypt; ^2^College of Agronomy, Anhui Agricultural University, Hefei, China; ^3^National Key Lab of Crop Genetic Improvement, Huazhong Agricultural University, Wuhan, China; ^4^Department of Biology, Faculty of Science, University of Tabuk, Tabuk, Saudi Arabia; ^5^Department of Plant Agricultural, Faculty of Agriculture Science, Al-Azhar University, Assiut, Egypt; ^6^Plant and Soil Sciences Division, Davis College of Agriculture, Natural Resources, and Design, West Virginia University, Morgantown, WV, United States; ^7^Department of Plant and Soil Sciences, Institute for Agricultural Biosciences, Oklahoma State University, Ardmore, OK, United States; ^8^State Key Laboratory of Tea Plant Biology and Utilization, College of Tea and Food Science and Technology, Anhui Agricultural University, Hefei, China

**Keywords:** *Glycine max*, *Bradyrhizobium japonicum*, root growth and nodulation, strigolactone, terpenoid synthesis gene

## Abstract

In legumes, many endogenous and environmental factors affect root nodule formation through several key genes, and the regulation details of the nodulation signaling pathway are yet to be fully understood. This study investigated the potential roles of terpenoids and terpene biosynthesis genes on root nodule formation in *Glycine max*. We characterized six terpenoid synthesis genes from *Salvia officinalis* by overexpressing *SoTPS6, SoNEOD, SoLINS, SoSABS, SoGPS*, and *SoCINS* in soybean hairy roots and evaluating root growth and nodulation, and the expression of strigolactone (SL) biosynthesis and early nodulation genes. Interestingly, overexpression of some of the terpenoid and terpene genes increased nodule numbers, nodule and root fresh weight, and root length, while others inhibited these phenotypes. These results suggest the potential effects of terpenoids and terpene synthesis genes on soybean root growth and nodulation. This study provides novel insights into epistatic interactions between terpenoids, root development, and nodulation in soybean root biology and open new avenues for soybean research.

## Introduction

Soybean nodulation occurs through symbiotic interactions between root cortical cells and the soil bacteria, *Bradyrhizobium japonicum*, leading to the formation of novel structures called root nodules. Rhizobia are hosted inside the specialized root nodule and fed carbohydrates and all nutrients necessary to maintain their lives and, in return, convert atmospheric nitrogen into ammonia, which is available for the plant (Ferguson et al., 2005). Root nodule formation is strictly controlled locally and systemically by several plant hormones and metabolic genes during nodule initiation, nodule development, and active nitrogen fixation. Furthermore, several genes related to specialized metabolisms in legumes (e.g., terpenoid and phenylpropanoid biosynthesis) were identified by microarray analysis in *Lotus japonicus* nodules with higher expression levels in the nodule parenchyma and the nodule vascular bundle compared with the infection zone (Takanashi et al., 2012). Accordingly, the knockdown of the gene 3-Hydroxy-3-Methylglutaryl Coenzyme-A Reductase1 (*MtHMGR1*) in *Medicago truncatula* led to a dramatic decrease in nodulation, confirming that a key enzyme regulating the Mevalonate (MVA) pathway, *MtHMGR1*, interacts with *DMI2* and is required for nodule development (Kevei et al., 2007; Venkateshwaran et al., 2015). Moreover, the gene silencing of *GmMAX1a* and *GmMAX4a* by RNA interference (RNAi) also resulted in a drastic reduction in nodule numbers (Haq et al., 2017; Rehman et al., 2018; Ahmad et al., 2020).

Terpenoids encompass the largest class of specialized metabolites with different structures found predominantly in plants (Pott et al., 2019). As one group of medicinal plants used worldwide, Salvia species produce various terpenoid compounds with highly diverse structures that can play important functions in fundamental biological processes (Degenhardt et al., 2009; Ali et al., 2017, 2018). Croteau and coworkers first elucidated the reaction mechanism for terpenoid synthases by studying native enzyme reactions with substrate inhibitors, analogs, and intermediates (Korankye et al., 2017). However, the exact functions of the genes involved in terpenoid biosynthesis and their biological roles in Salvia species and soybean (*Glycine max*) remain unknown.

Researchers recently identified a terpene synthase (TPS) that can convert one carbocation to a mixture of others through various cyclizations, rearrangements, or hydride shifts (Luck et al., 2020). Thus, TPS enzymes have the unique catalysis ability to convert intermediate substrates into a single or several products during different reaction cycles (Zhou and Pichersky, 2020). *S. officinalis* (+)-sabinene synthases produced *in vitro* 63% (+)-sabinene, 21% γ-terpinene, 7% terpinolene, 6.5% limonene, and 2.5% myrcene (Ali et al., 2017). Five *S. officinalis* TPS genes expressed in tobacco generated different types and amounts of mono-, sesqui-, di-, triterpenes, and other terpenoid compounds (Ali et al., 2017). Studies on *S. guaranitica* also revealed the production of primarily monoterpenes and a large amount of sesquiterpenes (Ali et al., 2018). Three-dimensional structural analysis of some plant TPS, such as a sesquiterpene synthase from *Nicotiana tabacum* (Starks et al., 1997) and three monoterpene synthases, respectively, from *Salvia officinalis* (Whittington et al., 2002), *Mentha spicata* (Hyatt et al., 2007), and *Salvia fruticosa* (Kampranis et al., 2007) revealed extensive similarities despite differences in the reaction mechanism, which depends on the amino acid residues forming the active site cavity, the type of ions required for catalysis, and the type and number of protein terminal domains and conserved motifs (Degenhardt et al., 2009).

The metabolism of isoprenoids is also involved in the biosynthesis of plant hormones, such as gibberellic acids (GAs), cytokinins (CKs), strigolactones (SLs), brassinosteroids (BRs), and abscisic acid (ABA) (KEKK).^1^ These phytohormones fundamentally regulate plant growth and development, such as stem growth (tropism), branching, leaf shape, ripening, root architecture, and root nodulation (Kildegaard et al., 2021). Particularly, these hormones essentially affect root-rhizobia interaction, nodule organogenesis and development (Ryu et al., 2012; Breakspear et al., 2014; Ferguson and Mathesius, 2014; Waldie et al., 2014). GAs are terpenoids formed from geranylgeranyl diphosphate (GGDP) after a series successive oxidation steps catalyzed by a number of GA oxidases (Sun et al., 2006). Furthermore, all isoprenoid CKs, such as N^6^-isopentenyladenine and trans-zeatin, are accumulated upon rhizobia infection or Nod factor treatment, and CKs have central role in nodule organogenesis (van Zeijl et al., 2015). In addition, SLs are also derived from carotenoid broken-down and been implicated in legume nodulation (van Zeijl et al., 2015; Haq et al., 2017; McAdam et al., 2017; Rehman et al., 2018; Ahmad et al., 2020). Although SL biosynthesis and signaling have been studied in Arabidopsis, rice, pea, and petunia, there are only a few reports on SLs in soybean (Haq et al., 2017; Rehman et al., 2018; Ahmad et al., 2020). Recent studies suggested that diverse and parallel SL biosynthesis pathways and signal transduction mechanisms may exist (Waldie et al., 2014). Indeed, various hormones, including auxin, GAs, jasmonate (JA), BRs, and ABA, are involved in regulating legume-rhizobia interaction and nodulation, and impact SL biosynthesis and signaling in legumes (Breakspear et al., 2014; Ferguson and Mathesius, 2014). SLs, in turn, affect auxin, JA, ABA biosynthesis and transport and thereby modulate bud outgrowth and root development (Suzuki et al., 2011; Shinohara et al., 2013). However, the complex hormone crosstalk’s remain to be fully understood for nodule development and symbiotic nitrogen fixation. Therefore, it is of great relevance to examine how terpenoid pathways interact with other hormones and affect root development and nodulation in legumes. Here, we characterized six genes from *S. officinalis* involved in terpenoid and terpene biosynthesis to determine the impact of overexpressing terpenoid biosynthesis genes on rhizobial interaction and nodulation in soybean roots. Since these genes all involved in terpenoid pathways or metabolic networks, they might influence each other. For that, we (i) overexpressed those genes in soybean hairy roots; (ii) examined root and nodulation phenotypes at 10 and 20 days post-inoculation with *B. japonicum* (USDA110); and (iii) analyzed the expression of genes involved in the terpenoid, terpene, strigolactones biosynthesis and nodulation signaling pathways via qRT-PCR. Altogether, our data provide novel insights into the epistatic relationships between terpenoids, strigolactones biosynthesis, and the nodulation signaling pathway.

## Materials and Methods

### Bioinformatics Prospection of Terpenoid Biosynthetic Genes

For phylogenetic analysis and identification of terpenoid biosynthesis genes in the *Glycine max* genome, we searched the RNA-Seq Data Analysis and Phytozome database^2^ and identified soybean proteins with high sequence similarity (≥90% normalized identity) to annotated terpenoid biosynthesis genes of *S. officinalis* through BlastP. A phylogenetic tree of terpenoid biosynthesis genes was constructed with MEGA6 using the Neighbor-Joining method with 1,000 bootstraps. Tissue-specific expression data from nine soybean tissues (seed, root, hairy root, nodule, stem, leaf, flower, shoot apical meristem, and pods) were extracted from public RNA-Seq data databases (see text footnote 2). Putative subcellular localization of terpenoid biosynthesis gene products from *S. officinalis* was inferred from their sequence similarity to characterized Arabidopsis enzymes at the Arabidopsis Information Resource.^3^ Subcellular localization profile images were built using Cell Electronic Fluorescent Pictograph Browsers (Cell eFP).^4^

### Plant Material and Tissue Collection

*Salvia officinalis* seeds were kindly provided by the staff member of Egyptian Desert Gene Bank (EDGB) of Desert Research Center (DRC), Egypt. Seed were grown in a growth chamber at Huazhong Agricultural University (HZAU) set at 22°C day/20°C night with 60–70% humidity and a 16-h photoperiod with 100–150μmol?m^–2^ s^–1^ light density using fluorescent bulbs. All samples were collected and snap-frozen in liquid nitrogen and then stored at –80°C until RNA extraction.

### RNA Extraction and cDNA Synthesis

Total RNA from three biological replicates *S. officinalis* was extracted from leaves using TRIzol reagent (Invitrogen). Similarly, total RNA was extracted from ten to twelve biological replicates of *G. max* hairy roots and nodulating roots upon 10 and 20 days of rhizobial inoculation. Total RNA samples were treated with DNase I (Takara). RNA quality was examined on 1% Agarose gels, and the purity and concentration were analyzed using a Nano-Photometer spectrophotometer (IMPLEN, CA, United States). cDNA synthesis for gene cloning and qRT-PCR was performed with a 10 μg total RNA pool produced by mixing equal volumes of the three RNA replicates in a tube using a commercial reverse transcription kit (M-MLV, China) according to the manufacturer’s protocol.

### Cloning of Full-Length Terpenoid Synthase cDNAs

Full-length cDNAs for *SoTPS6, SoNEOD, SoLINS, SoSABS, SoGPS*, and *SoCINS* were obtained by PCR amplification with short and long gene-specific primers designed based on the transcriptome sequencing of *S. officinalis* leaves (Supplementary Table 1). Leaf cDNA was used as a template for the first PCR, which was performed with short primers and the KOD-Plus DNA polymerase (Toyobo, Japan) with the following cycling conditions: an initial step of 3 min at 94°C followed by 35 cycles of denaturation for 10 s at 98°C; 30 s at 60, 57, 60, 58, 60, and 60°C (different annealing temperatures for each respective gene) and an extension for 1.5 min at 68°C, and a final extension step for 10 min at 68°C. The first PCR products were used as templates for PCR cloning using long primers and KOD-Plus DNA polymerase. The amplified PCR products were purified using (QIAEX II Gel Extraction Kit, China) and cloned into the Gateway entry vector pDONR221 using BP Clonase (Gateway™ BP Clonase™ II Enzyme mix, Invitrogen). The resulting pDONR221 constructs harboring target genes were sequenced, and the LR Clonase (Gateway™ LR Clonase™ Enzyme mix, Invitrogen) was used for recombination into the destination vector pB2GW7 for *G. max* hairy root transformation to produce composite soybean plants. Sanger sequencing confirmed that all final constructs contained *SoTPS6, SoNEOD, SoLINS, SoSABS, SoGPS*, and *SoCINS*. The constructs were introduced into *Agrobacterium rhizogene*s strain “K599” by direct electroporation.

### Soybean Hairy Root Transformation and Rhizobial Inoculation

Seeds of soybean cv. “Tianlong” were surface sterilized by placing 150 seeds in 15 × 100 mm Petri dishes in a single layer. The plates were placed inside a 1,000-mL beaker with 200 mL of commercial bleach. Ten micro-liters of concentrated (12 N) HCl were applied drop wise to the beaker’s internal wall, the container was sealed with a plastic cover and kept overnight (16 h). The following morning, the sterilized seeds were put to germinate in sterilized vermiculite in a growth chamber (12-h photoperiod, 28°C in the day/25°C at night, and 70% humidity) for a few days until hairy root transformation.

Recombinant *A. rhizogene*s were grown for 2 days at 28°C on solid LB media supplemented with 50 μg/mL of each streptomycin and spectinomycin. An individual colony of each construct was inoculated into 1 mL of liquid LB medium with the same antibiotics and grown at 28°C under 200 rpm agitation overnight. After 24 h, the liquid cultures were transferred into a 250-mL conical flask containing 50 mL of LB media supplemented with the same antibiotics and grown in a shaker at 28°C until an optical density (OD_600_) of 0.6–8.0 was reached. Overnight cell cultures were harvested by centrifugation at 5,000 rpm for 10 min at 4°C, and the pellet was re-suspended to an OD of half-strength B5 medium containing 3% sucrose. Healthy and vigorous seedlings with unfolded green cotyledons were inoculated with *A. rhizogenes* strain K599 harboring the binary vectors by injecting the hypocotyls proximal to the cotyledon with the bacterial suspension. The infected seedlings were then transplanted into a 10 cm × 10 cm × 8.5 cm pots with vermiculite with the infection site buried, and each pot was covered with a transparent plastic bag to retain humidity.

The rhizobial inoculations of hairy roots were carried out with 15-day old plants (10 days after root transformation). A culture of *B. japonicum* strain “USDA-110” was cultured onto yeast extract mannitol agar (YMA) at 28°C. After 10 days of hairy root emergence, the optical density (OD_600_) of a rhizobium liquid YM culture was adjusted to 0.08–1.0, and about 50 mL were applied to each pot. After 10 and 12 days of rhizobial inoculation, the plants (*n* = 10–12) with well-developed hairy roots and nodules were photographed and harvested for measurements and RNA isolation to assess gene expression. Hairy root systems from each plant were considered independent transformation events.

### Quantitative Real-Time PCR Analyses

Quantitative real-time PCR (qRT-PCR) was performed using an iQ™5 Multicolor Real-Time PCR Detection System (Bio-Rad) with SYBR Green fluorescence (and ROX as a passive reference dye; Newbio Industry, China) in a total reaction volume of 20 μL, as described previously (Ali et al., 2017, 2018). Gene-specific primers for *GmActin* as a reference gene and the terpenoid biosynthesis genes under study (*SoTPS6, SoNEOD, SoLINS, SoSABS, SoGPS*, and *SoCINS*) were used. Primers were designed with the IDTdna tool,^5^ and their sequences are listed in Supplementary Table 2. Additionally, gene-specific primers for seventeen *G. max* genes involved in Strigolactone biosynthesis and nodulation signaling pathway (*GmMAX1*α, *GmMAX1*β, *GmMAX2, GmMAX3, GmMAX4α, GmMAX4β, GmNINα, GmNINβ, GmNRF5, GmNSP1α, GmNSP1β, GmNSP2α, GmNSP2β, GmDMI2α, GmDMI2β, GmDMI3*α, and *GmDMI3*β) are listed in Supplementary Table 3. The amplicon sizes were designed between 146 and 160 bp. The quantitative RT-PCR standard conditions were: 95°C for 3 min, 35 amplification cycles (95°C for 10 s, 58°C or 60°C for 30 s, and 72°C for 20 s), followed by 65°C for 5 s and 95°C for 5 s). The relative expression levels were calculated by comparing the cycle thresholds (CTs) of the target genes with that of the reference gene *GmActin* using. Data quantification was carried out with the Bio-Rad IQ™ 5 Multicolor Real-Time Manager software using the 2^–ΔΔ*Ct*^ method (Ali et al., 2018) and *GmActin* as a reference housekeeping gene for normalization. Values are presented as means ± SE of three different RNA pool replicates.

### Metabolite Extraction From Transgenic Soybean Hairy Roots

Terpenoid compounds from *in vitro* transgenic soybean hairy root containing either GUS as a control and transgenic soybean hairy root containing the *SoTPS6, SoNEOD, SoLINS, SoSABS, SoGPS*, and *SoCINS* expression constructs were extracted and isolated. For this, ten grams from each transgenic hairy root line (one gram from each replicate) were homogenized in liquid nitrogen with a mortar and pestle, after which the macerate was directly soaked in n-hexane as a solvent into 60 mL amber storage bottles with screw-top vials and silicone/PTFE septum lids (Sigma-Aldrich) to reduce volatile losses to the headspace. Samples were then incubated with shaking at 37°C and 200 rpm for 72 h. Subsequently, the solvent was transferred using a glass pipette into a 10-mL glass centrifuge tube with screw-top vials and silicone/PTFE septum lids and centrifuged at 5,000 rpm for 10 min at 4°C to remove plant debris. The supernatant was pipetted into screw-cap glass vials, and the material was concentrated to 1.5 mL of concentrated oils under a stream of nitrogen gas with a nitrogen evaporator (Organomation) in a water bath at room temperature (Toption-China-WD-12). The concentrated oils were transferred to a fresh crimp vial amber glass, 1.5 mL screw-cap vials with silicone/PTFE septum lids. For complete oil recovery, the remaining film crude oil in the internal surface of glass vials where the material was concentrated was dissolved in a minimum volume of n-hexane, thoroughly mixed, and transferred to the same fresh crimp 1.5-mL vial amber glass. Each crimp vial was then placed on the auto-sampler of the gas chromatography-mass spectrometer (GC-MS) system for analysis. Alternatively, each tube was covered with parafilm after closing with screw tops with silicone/PTFE septum lids and stored at –20°C until analysis (Ali et al., 2017, 2018).

### Gas Chromatography-Mass Spectrometer Analysis of Essential Oil Components

GC analysis was performed using a Shimadzu model GCMS-QP2010 Ultra (Tokyo, Japan) system. An approximately 1-μL aliquot of each sample was injected (split ratios of 15:1) into a GC-MS equipped with an HP-5 fused silica capillary column (30 m × 0.25 mm ID, 0.25 μm film thicknesses). Helium was used as the carrier gas at a constant flow of 1.0 mL/min^–1^. The mass spectra were monitored between 50 and 450 *m/z*. The temperature was initially set under isothermal conditions at 60°C for 10 min, thereafter the temperature increased at a rate of 4°C/min^–1^ to 220°C, held isothermal at 220°C for 10 min, increased by 1°C/min^–1^ to 240°C, held isothermal at 240°C for 2 min, and finally programmed to 350°C at a rate of 5°C/min and kept constant at 350°C for 10 min. The identification of the volatile constituents was determined by parallel comparison of their recorded mass spectra with the data stored in the Wiley GC/MS Library (10th Edition; Wiley, New York), the Volatile Organic Compounds (VOC) analysis S/W software, and the NIST library (2014 edition). The relative% amount of each component was calculated by comparing average peak areas to the total areas. All experiments were performed simultaneously three times under the same conditions for each isolation technique with a total GC running time of 80 min (Ali et al., 2017, 2018).

### Statistical Analyses

Soybean hairy root measurements were analyzed by the Student’s *t*-test to estimate the effects of gene overexpression and time on the number of nodules, nodule fresh weight (gram), fresh root weight (gram), and root length (cm) compared to the control roots (*GUS*-overexpressing hairy roots). Each column represents the mean ± SD of the parameter, and statistical significance was based on the Student’s *t*-test (*P < 0.05 and ^**^P < 0.01) with GUS-overexpressing hairy roots as control.

**Gene accession numbers:** *Salvia officinalis* (-)-germacrene D synthase (*SoTPS6*, KY399783.1); (+)-neomenthol dehydrogenase (*SoNEOD*, KY399785.1); (3S)-linalool synthase (*SoLINS*, KY399786.1); Sabinene synthase (*SoSABS*, KY399785); Geranyl-diphosphate synthase (*SoGPS*, KY399788); 1,8-cineole synthase (*SoCINS*, KY399782).

## Results

### Identification of Terpenoid Biosynthesis Genes From Sage Plants

In order to identify the putative terpenoid biosynthetic genes in the soybean genome, a BLASTP search against the soybean genome was conducted using functionally characterized *S. officinalis* terpenoid biosynthesis proteins as queries. This approach identified several proteins closely related to *SoTPS6, SoNEOD, SoLINS, SoSABS, SoGPS*, and *SoCINS.* These sequences were submitted to phylogenetic analysis (Supplementary Figure 1). The expression patterns of putative terpenoid biosynthesis genes of soybean were uncovered by transcript analysis across nine tissues (pod, leaf, root hair, root, nodule, seed, shoot apical meristem, stem, and flower) using the Phytozome database (see text footnote 2). Expression levels of *Glyma.07G073800.2* gene was highest in flower then leaves, seeds, stem, pod, sam, nodules, root and root_hairs. Moreover, *Glyma.05G100400.4* transcription levels was markedly increased in leaves, root, root_hairs, nodules, flower, seed, sten, sam and pod. Furthermore, expression levels of *Glyma.03G014300.1* gene was highest in stem, seed, flower, leaves, sam pod, nodule, root and root_hairs. While, the *Glyma.07G074600.2* was highly expressed at seeds, roots, sam, root_hairs, nodules, pods, leaves, flowers and stems. At the end, the other genes were expression with different levels at various tissues (Supplementary Figure 2).

In plants, there are two pathways responsible for terpene biosynthesis: the plastidial (MEP, methylerythritol 4-phosphate) pathway that generates monoterpenes, diterpenes, and carotenoids, and the cytosolic (MVP, mevalonate) pathway that produces sesquiterpenes, triterpenes, and sterols (Singh and Sharma, 2015; Pazouki and Niinemets, 2016). A correlation also exists between terpene, SLs, and nodulation signaling molecules since all of them are derived from terpenoids/isoprenoids. Co-expression and subcellular localization analyses can also be helpful to identify connections between different processes, such as terpenoid biosynthesis, SLs derived from carotenoid catabolism, and the nodulation signaling pathway in soybean. Therefore, we further explored the potential sub-cellular localization of the gene products from S. officinalis based on Arabidopsis protein localization to recognize possible synthesis sites using the Cell eFP browsers (see text footnote 4). From this analysis, the terpenoid biosynthesis genes localize mainly to the cytosol, mitochondrion, nucleus, and plastid (Supplementary Figure 3).

### Overexpression of Terpenoid Genes Changed Soybean Root Growth Without *B. japonicum* Infection

**FIGURE 1:**
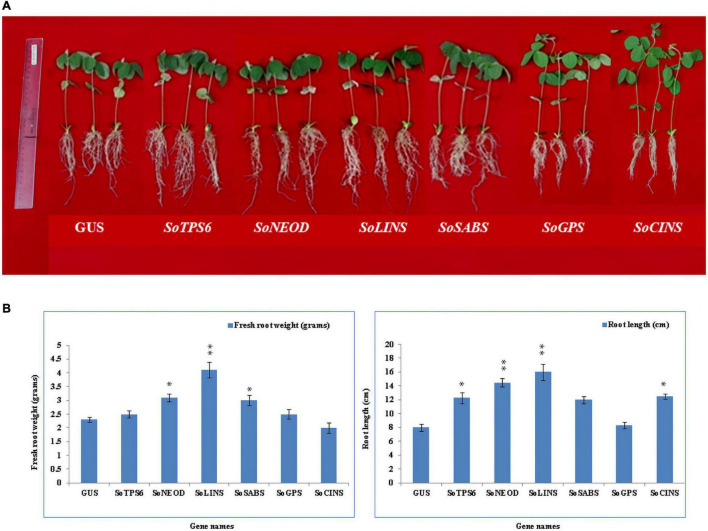
Effects of terpenoid gene overexpression on root and shoot growth. **(A)** Representative photos of chimerical *G. max* plants with transgenic hairy roots overexpressing terpene synthesis genes. Chimerical soybean plants were generated by transformation with K599 harboring terpene genes including (left to right), GUS (control), *SoTPS6, SoNEOD, SoLINS, SoSABS, SoGPS*, and *SoCINS*. **(B)** *In vivo* hairy roots’ fresh weight (gram) and root length (cm). Root phenotypes were examined for at least 10 independent lines (*n* = 10). Each column represents the mean ± SD of the parameter and statistical significance was based on the Student’s *t*-test (*P < 0.05; ^**^ *P* < 0.01; (n.s.), not significant) with GUS-overexpressing hairy roots as control.

In order to test the effect of terpenoid biosynthesis genes on soybean root phenotypes, composite *G. max* plants harboring hairy roots overexpressing terpene genes *SoTPS6, SoNEOD, SoLINS, SoSABS, SoGPS*, and *SoCINS* were compared to control hairy roots overexpressing *GUS*. First, non-inoculated (non-nodulating) 25-day-old hairy roots grown in vermiculite were assessed. Surprisingly, overexpression of *SoNEOD, SoLINS* and *SoSABS* genes studied led to significantly higher root fresh weight than *GUS* as a control. While, overexpression of *SoTPS6, SoNEOD, SoLINS*, and *SoCINS* genes led to significantly longer roots than *GUS* control (Figures 1A,B). These results suggest that terpenes play a decisive role in soybean root development (Figures 1A,B). In line with our results, previous reports found that terpene metabolism in roots was a highly coordinated, cell-specific process (Tholl, 2015). Other reports demonstrated that thalianol and marneral triterpene biosynthetic gene clusters were co-expressed primarily in the root epidermis (Field and Osbourn, 2008; Field et al., 2011). Moreover, Kemen et al. (2014) determined the role of β-amyrin synthesis as a triterpene synthesized in root epidermal cells of oats that produce a “super hairy” root phenotype. Several TPS family genes in Arabidopsis were expressed in different root tissues (Ro et al., 2006; Wang et al., 2016), such as the rhizathalene diterpene synthase (*AtTPS08*) primarily expressed in the root stele (Vaughan et al., 2013). Furthermore, two 1,8-cineole monoterpene synthase genes from Arabidopsis were constitutively expressed in the stele of the root elongation and differentiation/maturation zones as well as in the epidermis and cortex of older roots (Chen et al., 2011). A similar expression pattern was observed for two closely related (Z)-γ-bisabolene sesquiterpene synthases (Chen et al., 2004). These results underscore the role of terpenoid biosynthesis in root development and open new avenues for soybean research.

### Essential Oils Produced in Transgenic Soybean Hairy Roots Overexpressing Terpene Synthetic Genes

To functionally characterize terpenoid biosynthesis genes in transgenic *G. max* hairy roots, six terpenoid biosynthesis genes (*GUS* as a control, *SoTPS6, SoNEOD, SoLINS, SoSABS, SoGPS*, and *SoCINS*) from *S. officinalis* were cloned and evaluated for their potential to modulate terpenoid production in transgenic *G. max* hairy roots. The qualitative and quantitative analyses of all compounds from the essential oils are reported in Table 1. In roots overexpressing *SoTPS6*, sesquiterpenes represented the main compounds (11.78%), followed by monoterpenes (10.78%) and one diterpene compound (10.17%). Moreover, in roots overexpressing *SoCINS* diterpenes were the major group accounting 11.05, followed by monoterpenes (10.16%), and sesquiterpenes (7.19%). Meanwhile, roots overexpressing *SoGPS* produced monoterpene (3.04%), followed by sesquiterpenes (1.39%) and one diterpene compound (0.43%). On the other hand, monoterpenes (5.0%) were observed as the major compound category in roots overexpressing *SoSABS*. followed by one sesquiterpenes (0.93%). Furthermore, roots overexpressing *SoNEOD* produced monoterpene (3.13%), followed by one triterpenes (0.45%). Overall, the six hexane extracts from the different transgenic lines of *G. max* hairy roots showed unique and mutual metabolic features (Table 1).

**TABLE 1:** The major chemical composition and terpenes from transgenic *Soybean hairy* roots.

N	Compound name	R.T	Terpene type	*GUS*	*SoTPS6*	*SoNEOD*	*SoLINS*	*SoSABS*	*SoGPS*	*SoCINS*
1	(8) Annulene	5.238		9.54	0.97	5.94	10.41	0.3	2.34	0.35
2	Artificial Almond Oil	8.382	Organic						0.2	
3	Dihomo-γ-linolenic acid	9.448						0.6		
4	*Cis*-verbenol	10.786	Mono-	0.61	0.09	0.35		0.65		0.05
5	Dihydrocarveol	14.176	Mono-		0.11	0.37			0.22	
6	Limonene dioxide	16.262	Mono-				1.18			
7	Isomenthol	16.686	Mono-	3.12	0.5	2.08	3.23	3.43	0.78	0.44
8	Pinocarveol	17.503	Mono-						0.22	
9	α-Terpineol	19.305	Mono-						0.17	
10	Naphthalene	20.446				0.28				
11	Farnesan	24.283	Sesqui	0.41						
12	Dodecamethylcyclohexasiloxane	25.403			3.17				0.48	0.81
13	Farnesan	27.982	Sesqui							0.04
14	n-Cetane	28.864				0.19				
15	Caryophyllene oxide	31.027	Sesqui	0.67	11.78			0.93	1.06	7.15
16	4-Caranol	31.391	Mono-			0.33			0.75	
17	Isopulegol	35.93	Mono	0.67	10.08			0.92	0.54	9.67
18	Dihydrophytol	38.002	Diter							0.24
19	β-Carotene; β-Carotene, all-trans	39.66				0.61			0.35	
20	Stearic acid; n-Octadecanoic acid	42.285							0.58	
21	Linoleyl alcohol	42.974					2.98			
22	Erucic acid	44.418			0.5	11.93	9.79			
23	Palmitic acid	44.858		54.79	3.71	47.56	36.8	64.73	33.32	1.35
24	Ethyl palmitate	45.617								0.08
25	Cis, *cis-*linoleic acid	46.84		2.42			2.74	2.47	0.55	
26	Arachic acid	47.235				0.74		1.2		
27	Phytol	47.305	Diter		10.17				0.43	10.81
28	Adamantane, 1,3-dimethyl	48.167							0.19	
29	Oleic acid	49.096						10.89		
30	Stearic acid	49.545		1.56	0.56				7.21	0.26
31	γ-sitosterol	50.424		0.95	11.38		7.58	1.76		13.4
32	Mandelic acid	52.514					2.44			
33	Isopropyl linoleate	53.073				0.2				
34	Octadeamethyl-cyclononasiloxane	54.167		0.7	8.61			0.95	0.28	10.38
35	Oleyl amide	54.838		0.76	0.11	0.85		0.62		0.15
36	n-Heneicosane	56.512							0.43	
37	Methoprene	59.701		0.56	6.74			0.65	0.22	8.72
38	Pulegol	61.087	Mono-						0.36	
39	Phthalic acid dioctyl ester	62.27		1.6		1.39	1.64		0.44	
40	β,β-Carotene	63.348							0.14	
41	Octadeamethyl-cyclononasiloxane	67.034		0.53	6.17			0.65	0.2	8.1
42	Farnesane	72.172	Sesqui						0.33	
43	Squalene	78.212	Triter			0.45				
	Total percentage (%) of monoterpenes			4.4	10.78	3.13	4.41	5.0	3.04	10.16
	Total percentage (%) of sesquiterpenes			1.08	11.78			0.93	1.39	7.19
	Total percentage (%) of diterpenes				10.17				0.43	11.05
	Total percentage (%) of triterpenes					0.45				

### Overexpression of Terpenoid Biosynthesis Genes After Soybean Hairy Root Nodulation

**FIGURE 2:**
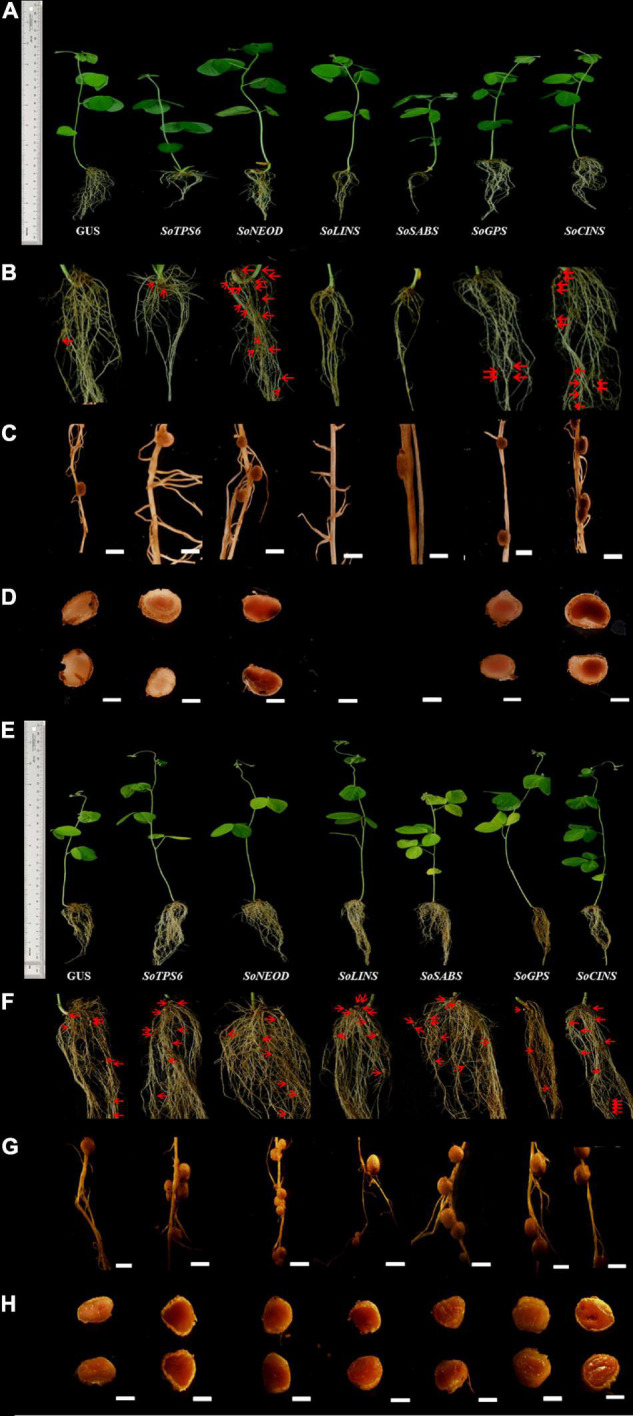
Effect of terpenoid gene overexpression on soybean root nodulation. Roots and nodules were examined at 10th and 20th day after rhizobia inoculated with *B. japonicum* strain USDA110. Composite plants were generated by transformation with the K599 vector harboring overexpression cassettes for *GUS* (control), *SoTPS6, SoNEOD, SoLINS, SoSABS, SoGPS*, and *SoCINS*. Roots were inoculated with rhizobia. **(A,E)** Root and shoot phenotypes of 10 and 20-days-old *G. max* plants. **(B,F)** Locations where nodules formed on hairy roots overexpressing 10 and 20-days after rhizobial inoculation. **(C,G)** Nodules developed on secondary roots. **(D,H)** Cross-sections of *G. max* nodules. Photographs in **(C,D,G,H)** were taken with a DP-73 microscope camera set (Olympus, Tokyo, Japan). Scale bars in **(C,D,G,H)** = 500 μm.

**FIGURE 3:**
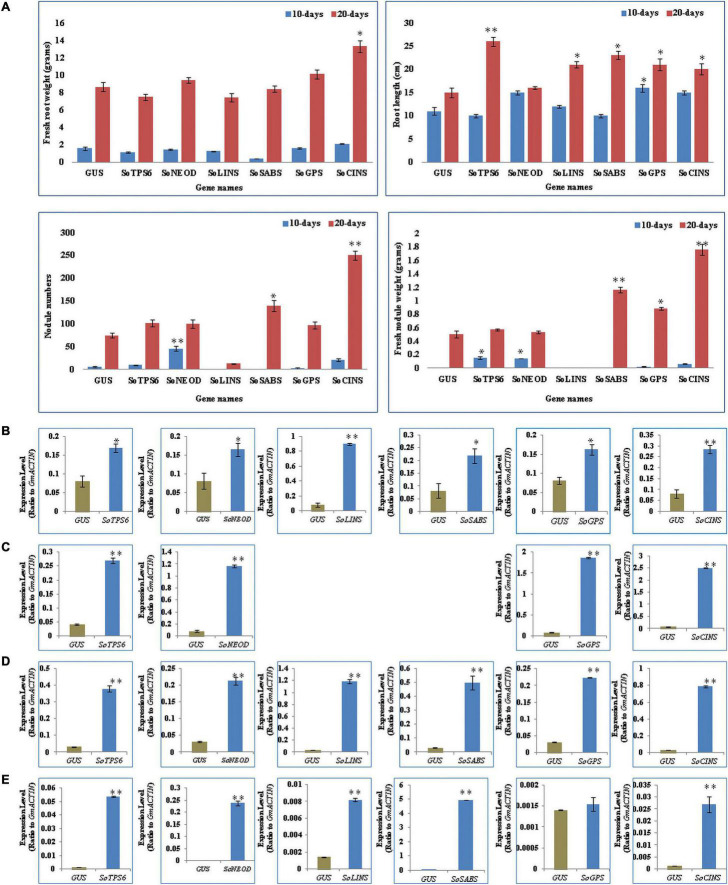
Effects of terpenoid synthesis gene overexpression on root growth and nodule development at 10 and 20 days of rhizobia inoculation. **(A)** *In vivo* fresh root weight (gram), and root length (cm), nodule numbers, fresh nodule weight (gram), were examined (*n* = 10–12). Blue and red columns represent the effect of gene overexpression after 10- and 20-days after rhizobial inoculation. Data is presented as means ± SD and statistical significance is based on Student’s *t*-test (*P < 0.05; ^**^P < 0.01) with GUS-overexpressing hairy roots as the control. **(B)** Quantitative RT-PCR for *in vivo* hairy roots after 10-days from *B. japonicum* (USDA110) infection. The error bars indicate the SD of three qRT-PCR biological replicates. **(C)** qRT-PCR for nodules after 10-days from infection. **(D)** qRT-PCR for *in vivo* hairy roots after 20-days from infection. **(E)** qRT-PCR for nodules after 20-days from infection.

Hairy roots of soybean overexpressing sage terpene biosynthesis genes were inoculated with *B. japonicum* (USDA110) and phenol-typed upon 10 days of rhizobial infection to explore the effects of terpenoid on soybean nodulation (Figures 2A–D) and root phenotypes (Figure 3A). The expression levels of each overexpressing gene in hairy roots were assessed via qPCR and compared to the GUS control hairy roots (Figures 3B,C). Our data clearly show that the overexpression of *SoTPS6*, *SoNEOD*, and *SoCINS* after 10 days from post-inoculation led to increase the nodule numbers and nodule fresh weight per root fresh weight compared to hairy roots overexpressing *GUS* (Figure 3A). The overexpression of *SoCINS* and *SoSABS*, however, caused reduced root growth and delayed the root nodulation, since few nodules were observed at 10 days post rhizobia inoculation. The hairy roots overexpressing *SoGPS*, *SoCINS*, and *SoNEOD* at 10 days post-inoculation were significantly longer than those with the *GUS* control (Figure 3A). We further checked these roots and nodules after 20 days of inoculation (Figures 2E–H). At 20 days post rhizobial infection, the expression of transgenes in hairy roots overexpressing *SoTPS6, SoNEOD, SoLINS, SoSABS, SoGPS*, and *SoCINS* was confirmed via qPCR (Figures 3D,E). The overexpression of terpenoid biosynthesis genes (*SoLINS* and *SoSABS*) after 20 days from infection increased the nodule numbers and root lengths compared to these phenotypes in 10-days-hairy roots. However, root length and nodule numbers in *SoLINS* and *SoSABS* lines were still lower than GUS control lines. In contrast, the overexpression of *SoCINS, SoSABS*, and *SoGPS* led to a significant increase in nodule fresh weight per root weight after 20 days from infection compared to either GUS (Figure 3A). Therefore, the effects of overexpressing terpenoid biosynthesis genes in hairy roots of soybean led to a sustained effect in nodule number and nodule fresh weight.

### Transgenic Soybean Hairy Roots Altered the Expression of Strigolactone Biosynthesis and Nodulation Genes

**FIGURE 4:**
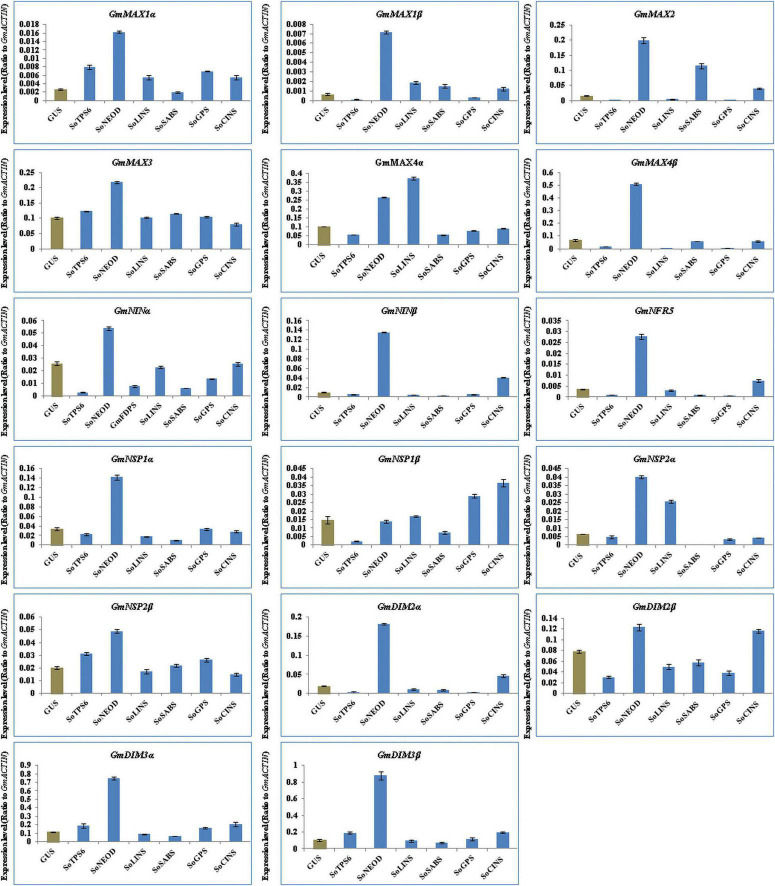
Expression profiles of SL biosynthesis and nodulation genes in soybean transgenic hairy roots after 10 days of rhizobia inoculation. Relative gene expression was analyzed using quantitative real-time PCR compared to GUS as a control. The housekeeping *GmB-ACTIN* gene was used as an internal reference gene for expression normalization. The error bars indicate the SD of three qRT-PCR biological replicates.

The function of GA and SLs on root nodulation in legumes has been well-documented in soybean, pea and lotus (Takaki et al., 2009; van Zeijl et al., 2015; McAdam et al., 2017; Rehman et al., 2018; Ahmad et al., 2020; Akamatsu et al., 2021). We examined the effect of terpenoid biosynthesis gene overexpression on the induction of SLs biosynthesis and signaling genes in hairy roots of soybean upon 10-day-post *B. Japonicum* infection to generate insights on the roles of terpenoids during symbiotic nitrogen fixation in legumes. Thus, we analyzed the expression levels of 17 selected genes, including SL synthetic genes such as *GmMAX1*α, *GmMAX1β, GmMAX2, GmMAX3, GmMAX4*α, and *GmMAX4*β, and early nodulation signaling genes such as *GmNINα, GmNINβ, GmNRF5, GmNSP1α, GmNSP1β, GmNSP2α, GmNSP2β, GmDMI2α, GmDMI2β, GmDMI3*α, and *GmDMI3*β. The results showed that the selected genes were differentially induced in hairy roots at 10 days post rhizobial infection (Figure 4). Expression levels of *GmMAX1*α, *GmMAX1β, GmMAX2, GmMAX3, GmMAX4β, GmNINα, GmNINβ, GmNRF5, GmNSP1α, GmNSP2α, GmNSP2β, GmDMI2α, GmDMI2β, GmDMI3*α, and *GmDMI3*β were highest in hairy roots overexpressing *SoNEOD*. *GmMAX4*α transcription levels was markedly increased in hairy roots overexpressing *SoLINS*, while the highest expression levels for *GmNSP1*β was observed in hairy roots overexpressing *SoCINS* (Figure 4). The expression patterns of SLs biosynthesis and nodulation signaling genes in hairy roots overexpressing terpenoid biosynthesis genes during the first 10 days of root nodulation suggest that, overall, terpenoid biosynthesis plays important, yet unknown, roles during nodulation signaling and the early stages of nodule development.

### Overexpressing Terpenoid Genes Changed the Expression of Strigolactone Biosynthesis and Nodulation Genes in Soybean Nodules

**FIGURE 5:**
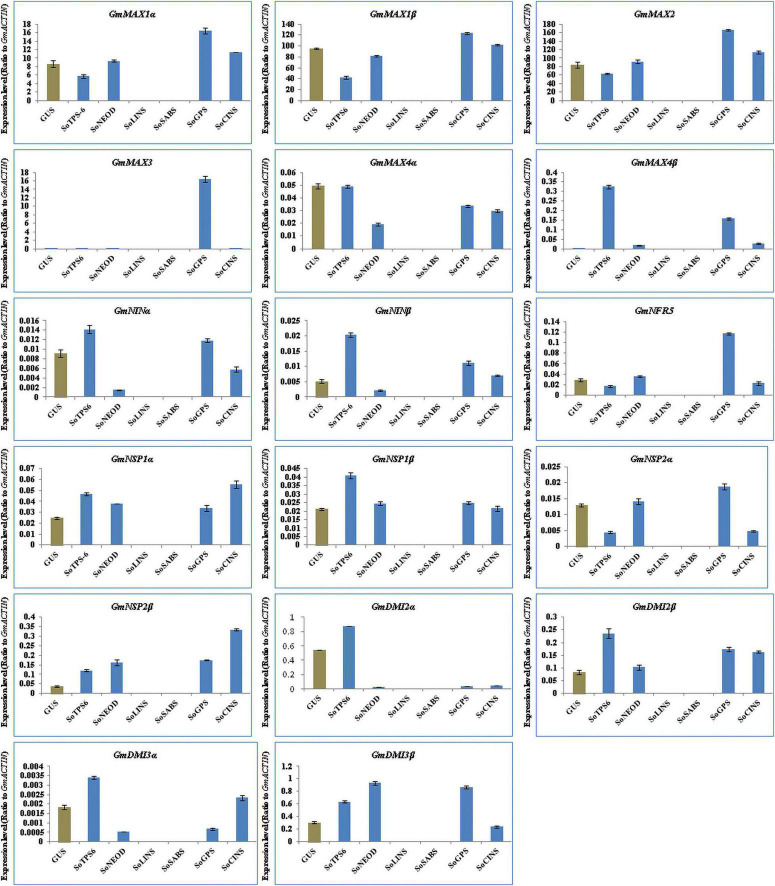
Expression profiles of SL biosynthesis and nodulation genes in soybean nodules after 10 days of rhizobia inoculation. Gene expression was analyzed using quantitative real-time PCR as compared to GUS as a control. The housekeeping *GmB-ACTIN* gene was used as an internal reference gene for expression normalization. The error bars indicate the SD of three qRT-PCR biological replicate.

To unveil the function that terpenoids play during the early stages of nodule formation, we studied the effects of expressing terpenoid biosynthesis genes on the expression of SLs biosynthesis and signaling genes in nodulation 10 days after *B. japonicum* infection. Therefore, we examined and analyzed the expression levels of the same previously selected genes, SL biosynthesis genes, and early nodulation signaling genes. The results showed that the selected genes were differentially induced in nodules at 10 days post rhizobial infection (Figure 5). Expression of *GmMAX1*α, *GmMAX1β, GmMAX2, GmMAX3, GmNRF5*, and *GmNSP2*α were highest in nodules overexpressing *SoGPS*. On the other hand, *GmMAX4β, GmNINα, GmNINβ, GmNSP1β, GmDMI2α, GmDMI2*β, and *GmDMI3*α transcription levels were markedly increased in nodules by overexpressing *SoTPS6*. Furthermore, expression of *GmNSP1*α and *GmNSP2*β were highest in nodules overexpressing *SoCINS.* Moreover, *GmDMI3*β expression was highest in nodules overexpressing *SoNEOD* (Figure 5). The expression patterns of SLs biosynthesis and nodulation signaling genes in nodules overexpressing terpenoid biosynthesis genes during the first 10 days of nodule formation suggest that, overall, terpenoid biosynthesis plays critical symbiotic roles during nodulation signaling and the early stages of nodule formation.

**FIGURE 6:**
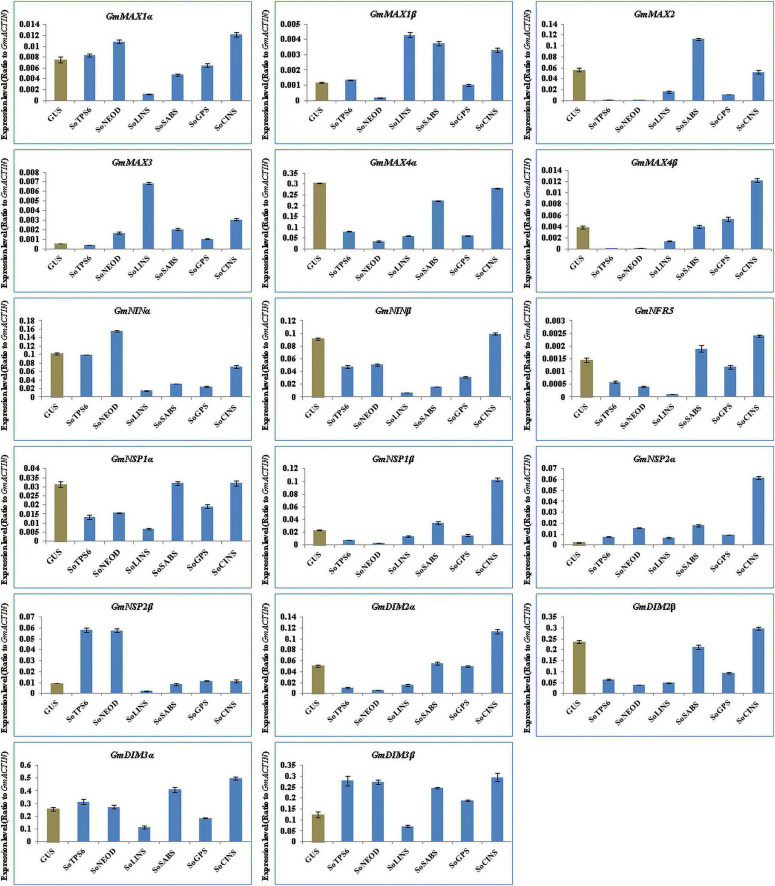
Expression profiles of SL biosynthesis and nodulation genes in soybean transgenic hairy roots after 20 days of rhizobia inoculation. Gene expression was analyzed using quantitative real-time PCR compared to GUS as a control. The housekeeping *GmB-ACTIN* gene was used as an internal reference gene for expression normalization. The error bars indicate the SD of three qRT-PCR biological replicates.

**FIGURE 7:**
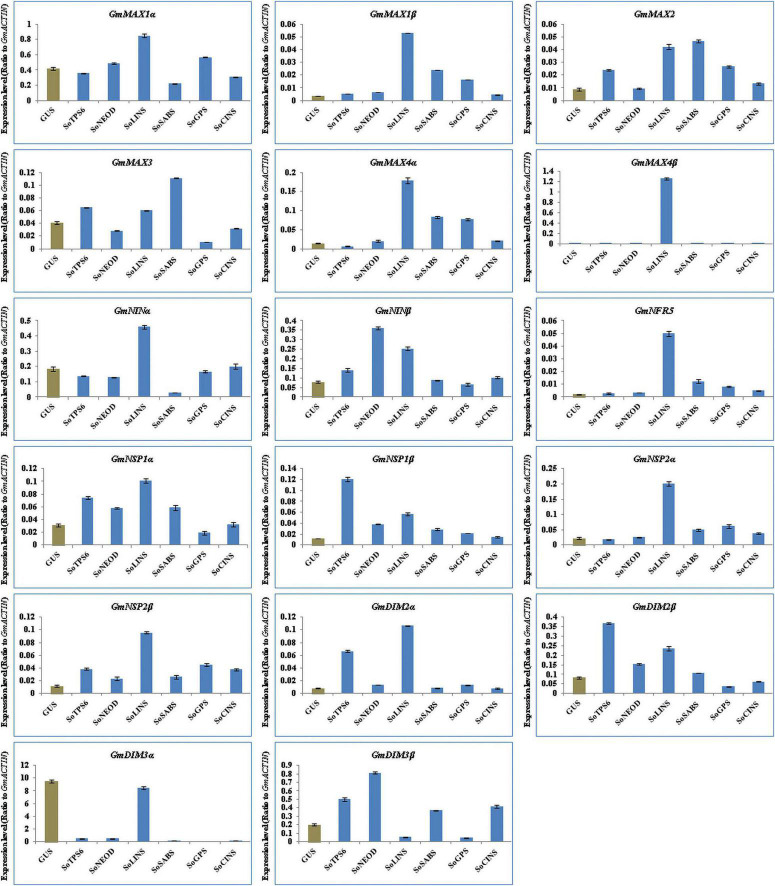
Expression profiles of SL biosynthesis and nodulation genes in soybean nodules after 20 days of rhizobia inoculation. Gene expression was analyzed using quantitative real-time PCR compared to GUS as a control. The housekeeping *GmB-ACTIN* gene was used as an internal reference gene for expression normalization. The error bars indicate the SD of three qRT-PCR biological replicates.

We further explored the effect of terpenoid biosynthesis gene overexpression on the expression of SLs biosynthesis and nodulation signaling genes in hairy roots and nodules of soybean at 20 days post-rhizobial infection to understand the sustained effect on root development and nodulation. We examined and analyzed the expression levels for the same set of SL biosynthesis and early nodulation genes. In this context, our results showed that the selected genes were differentially induced in hairy roots and nodules at 20 days post-rhizobial infection (Figures 6, 7). For example, the expression of *GmMAX1*α, *GmMAX4β, GmNINβ, GmNRF5, GmNSP1β, GmNSP2α, GmDMI2α, GmDMI2β, GmDMI3*α, and *GmDMI3*β were highest in hairy roots overexpressing *SoCINS*, while the highest level of *GmMAX1*β and *GmMAX3* transcription were observed in hairy roots overexpressing *SoLINS*. Moreover, the expression levels of, *GmMAX2* was highest in hairy roots overexpressing *SoSABS*. Expression levels of *GmNSP2*β was highest in hairy roots overexpressing *SoTPS6.* Furthermore, *GmNINa* expression was highest in hairy roots overexpressing *SoNEOD* (Figure 6). In addition, expression of *GmMAX1*α, *GmMAX1β, GmMAX4α, GmMAX4β, GmNINα, GmNRF5, GmNSP1α, GmNSP2α, GmNSP2*β, and *GmDMI2*α were the highest in nodules overexpressing *SoLINS*. The highest expression levels for *GmMAX2* and *GmMAX3* were observed in nodules overexpressing *SoSABS*. Besides, *GmNIN*β and *GmDMI3*β expression were the highest in nodules overexpressing *SoNEOD*, while *GmNSP1*β and *GmDMI2*β transcription were highest in nodules overexpressing *SoTPS6* (Figure 7). Consequently, the expression patterns of SL biosynthesis and nodulation signaling genes in hairy roots and nodules overexpressing terpenoid biosynthesis genes at 20 days post-inoculation suggest that terpenoids have a sustained effect in root development and nodulation, including in mature root systems undergoing active symbiotic nitrogen fixation.

## Discussion

### Characterization of Terpenoid Genes From Soybean and Sage Plants

Soybean (*G. max*) is one of the oldest domesticated polyploid plants and is among the most important food crops in the world. Wild soybeans produce several types of mono- and sesquiterpenes, such as linalool, α-pinene, *trans*-ocimene, α-humulene, and (E,E)-α-farnesene at different concentrations in various tissues (Liu et al., 2014). However, the knowledge about the roles of terpene genes in soybean remains limited (Cheng-Ying et al., 2011; Kai et al., 2012; Yang et al., 2012; Rastogi et al., 2014). In our study, we identified genetic components of terpenoid biosynthesis in the soybean genome by searching sequences similar to those well characterized in the garden sage (*S. officinalis*, Lamiaceae) (Supplementary Figure 1), examined their expression patterns across various tissues and organs by using the Phytozome database (Supplementary Figure 2) and inferred the putative sub-cellular localization of sage genes based on Arabidopsis protein localizations using the Cell eFP Browser (Supplementary Figure 3). We further cloned terpenoid biosynthesis genes from garden sage, over-expressed them individually in hairy root systems of soybean, and evaluated the root terpenoid products, their effects on root architecture, growth, and nodulation. Substantial differences in root growth, root nodulation, expression levels of SL biosynthesis and nodulation signaling genes were observed.

### Ectopic Expression of Terpenoid Biosynthesis Genes Affected Root Growth and Nodulation

Interestingly, we found that the overexpression of these terpenoid genes affect root growth and nodulation. Particularly, terpenoid hormones, such as SLs, BRs, GAs, and ABA, and phenylpropanoid signaling molecules, such as isoflavones and flavonoids, were reported to affect nodulation in *Pisum sativum* and *Lotus japonicus* (Takanashi et al., 2012; Serova et al., 2019). The cytosolic mevalonic acid (MVA) pathway produces IPP via enzymatic isomerization to DMAPP, both of which serve as substrates for cytosolic farnesyl diphosphate (FPP) synthase (FPS) to form FPP. Whereas the plastidic methylerythritol phosphate (MEP) pathway directly produces both IPP and DMAPP for producing geranyl diphosphate (GPP) and GGPP by the plastidic enzymes GPP synthase (GPPS) and GGPP synthase (GGPPS), respectively (Chatzivasileiou et al., 2019; Pu et al., 2021). While FPP in the cytosol serves as a precursor for sterols, saponins, and brassinosteroids, plastidic GGPP is utilized for chlorophyll, carotenoid, strigolactone, abscisic acid, and GA biosynthesis. Independently, cytosolic sesquiterpene synthases use FPP, and plastidic mono- and diterpene synthases use GPP and GGPP, respectively, as substrates. However, plenty of evidence showed the metabolic interaction between the MVA and MEP pathways through exchanging the common intermediates, such as IPP and dimethylallyl diphosphate (DMAPP) (Wang et al., 2019). The metabolic flux through the MEP pathway often exceeds and exports to the MVA pathway (Volke et al., 2019). Trafficking of IPP is mediated by transporters on the envelope membranes of plastids (Andrade et al., 2017). However, it is generally believed that the MVA pathway contributes to the majority of sterol and saponin, BR synthesis, and the MEP pathway contributes more to carotenoid-derived strigolactone, GAs, and ABA (Gutensohn et al., 2013). Therefore, the overexpression of terpenoid synthesis, either in the cytosol or chloroplast casted effects on the phenotypes studied. It has been reported that β-amyrin can affect root hair patterning in oat and HMGR1 affected nodule development in *M. truncatula* transgenic roots, likely through modulating sterol or saponin synthesis (Kevei et al., 2007; Venkateshwaran et al., 2015). Also, the recruitment of HMGR1 by NORK may be required to produce specific isoprenoid moieties involved in modifying signaling proteins or isoprenoid signals, such as phytosterols and CKs (Kevei et al., 2007; Venkateshwaran et al., 2015). Therefore, it is not surprising that the overexpression of terpenoid synthesis, either in the cytosol or chloroplast could similarly affect the rood development and nodulation, despite of details underlying these effects remain to be investigated in the future.

### Effect of Overexpressing Terpenoid Biosynthesis Genes in Soybean Nodulation

In plants, terpenoid hormones including ABA, GAs, CKs, GLs, and BRs are generated via the MEP and MVA pathways, or derived from broken-down products of carotenoids such as SLs. All of them are involved in the regulating root growth and nodulation. Overexpression of the terpenoid biosynthesis genes could affect terpenoid metabolic fluxes toward the biosynthesis of these hormones, which eventually affect hormone levels and signaling pathways and nodulation.

Besides for the biosynthesis of essential phytohormones, MEP and MVA pathways also lead to the biosynthesis of secondary metabolites, including isoprenes, monoterpenes, sesquiterpenes, diterpenes, and triterpenoid compounds such as saponins and sterols. These compounds either are specialized metabolites for plant-environment interactions, plant-plant communications, or plant defenses against microbial pathogen infections and herbivore attacks, and against various abiotic stresses, from UV radiation to drought and temperature stresses. Overexpression of these terpenoid biosynthesis genes could affect the upstream or downstream terpenoid pathways and end products. These products could also affect rhizobia growth, infection, and nodulation processes, which is largely not understood yet. The negative effects of *SoLINS* and *SoSABS* overexpression in soybean roots on root growth and nodulation may have the causes on this aspect.

Our previous studies have shown that SLs are involved in soybean root development and nodulation (Haq et al., 2017; McAdam et al., 2017; Rehman et al., 2018; Ahmad et al., 2020). By taking advantages of these SL genes’ information, we tested effects of overexpression of terpenoid genes on SL pathway genes, and tried to correlated them. *GmNFR5* is a central nodulation signaling gene in soybean that specifically recognizes and binds to compatible, species-specific Nod factors produced by rhizobia (Zgadzaj et al., 2016). Nodule inception genes (*GmNINa* and *GmNINb*) are early key regulators of nodule organogenesis and infection thread formation (Schauser et al., 1999). Genes involved in Nod factor signal transduction, such as *GmDMI2α, GmDMI2β, GmDMI3α, GmDMI3β, GmNSP1α, GmNSP1β, GmNSP2*α, and *GmNSP2*β, trigger downstream factors, such as ENOD40 (Dessein et al., 2009; Ahmad et al., 2020). In general, the overexpression of terpenoid biosynthesis genes from garden sage led to increased transcription of SL biosynthesis and nodulation signaling genes. This finding points to terpenoids playing critical roles in root development and nodulation in legumes. Our results demonstrate that the overexpression of the genes studied here played significant roles in increasing nodulation and root growth compared to control hairy roots of soybean overexpressing *GUS*.

We propose that SLs and BRs are involved with the still elusive phenomenon of auto-regulation of nodulation (AON) through a role in promoting nodule formation and maintaining the nodule meristematic activity (Ferguson et al., 2005; McAdam et al., 2017). Chemically, SLs are sesquiterpenes and structurally related to terpenoids/isoprenoids (Pandey et al., 2016). Given the current limited understanding of SL effects on nodulation and the intersection of this hormone biosynthesis and the terpenoid pathway, we explored the effects of overexpressing genes of the terpenoid biosynthesis pathway in soybean hairy roots on the expression of genes involved in SL biosynthesis as well as the nodulation signaling pathway. In general, the SL biosynthesis genes *GmMAX1α, GmMAX1β, GmMAX2, GmMAX3, GmMAX4*α, and *GmMAX4*β showed high expression levels in roots and nodules, and their transcriptional activity increased when terpenoid biosynthesis genes were overexpressed in hairy roots of soybean. It has been reported that β-amyrin can affect root hair patterning in oat and HMGR1 affected nodule development in *M. truncatula* transgenic roots, likely through modulating sterol or saponin synthesis (Kevei et al., 2007; Venkateshwaran et al., 2015). Also, recruitment of HMGR1 by NORK may be required to produce specific isoprenoid moieties involved in modifying signaling proteins or isoprenoid signals, such as phytosterols and cytokinins (Kevei et al., 2007; Venkateshwaran et al., 2015). Therefore, as many studies indicated that the downstream di-, or tri- terpenoids in terpenoid pathways, including ABA, GAs, BRs, saponins, sterols, and carotenoid broken-down products, including SLs. As key metabolic enzymes-coding genes, the overexpression of them might influence whole terpenoid pathways or metabolic networks. Both upstream or downstream terpenoid hormones or end products of these terpene synthase genes reported here could have diverse effects on soybean root growth and nodulation. Particularly these hormones. Our lab previously studied SL biosynthesis and signaling pathway genes, which provide convenient tools for investigation of SLs first. However, we could not exclude that overexpression of these terpene synthase genes in soybean roots may also affect other terpenoid hormones and signaling, or metabolites affecting nodulation, which need to be investigated in future.

## Conclusion

In conclusion, our findings suggest that terpenoid biosynthesis genes play distinct roles in root development and nodulation. Besides the effects of the over-accumulation of terpenoid volatile compounds detected in their hairy roots of overexpressing terpenoid synthesis genes may also affect the biosynthesis of terpenoid hormones due to metabolic spill out. Thus, our study posited the possibility of involvement of the hormone SLs or BRs in the plant-rhizobial symbiosis in transgenic hairy roots, which is worthy of further investigation. We could not exclude other factors may also involved in the complex effects of overexpressing yepenoid synthesis genes on root growth and nodulation. Nevertheless, the characterizations of terpenoid-related genes in soybean roots will not only facilitate functional studies related to symbiotic nitrogen fixation in legumes but potentially also facilitate the development of biotechnology to enhance legume nodulation through metabolic engineering. To our knowledge, this is the first study exploring terpenoid biosynthesis genes on legume nodulation, and provides novel insights into the function of terpenoids in symbiotic nitrogen fixation, pointing to a potential biotechnological application in agriculture.

## Data Availability Statement

The datasets presented in this study can be found in online repositories. The names of the repository/repositories and accession number(s) can be found in the article/Supplementary Material.

## Author Contributions

MA and JZ conceived and designed the study. MA, LM, AA, and QH performed *in vivo* transgenic soybean hairy roots and other experiments. DD and SA performed the qRT-PCR analyses. MA wrote the manuscript. JZ, XW, VB, and MT revised the manuscript. All authors discussed the results, commented on the manuscript, and participated in the analysis of the data.

## Conflict of Interest

The authors declare that the research was conducted in the absence of any commercial or financial relationships that could be construed as a potential conflict of interest.

## Publisher’s Note

All claims expressed in this article are solely those of the authors and do not necessarily represent those of their affiliated organizations, or those of the publisher, the editors and the reviewers. Any product that may be evaluated in this article, or claim that may be made by its manufacturer, is not guaranteed or endorsed by the publisher.

## Footnotes

1
https://www.genome.jp/pathway/map01070+C04691

2
phytozome.jgi.doe.gov

3
https://phytozome.jgi.doe.gov/pz/portal.html#!info?alias=Org_Athaliana

4
http://bar.utoronto.ca/cell_efp/cgi-bin/cell_efp.cgi

5
https://eu.idtdna.com/scitools/Applications/RealTimePCR/
