# Supplementary material for: Overexpression of Terpenoid Biosynthesis Genes From Garden Sage (Salvia officinalis) Modulates Rhizobia Interaction and Nodulation in Soybean
Source: Front Plant Sci. 2021 Dec 23;12:783269. doi: 10.3389/fpls.2021.783269 (PMC8733304; doi:10.3389/fpls.2021.783269)
Supplement: Supplementary file 1 [file Presentation_1.PPTX]

## Slide 1
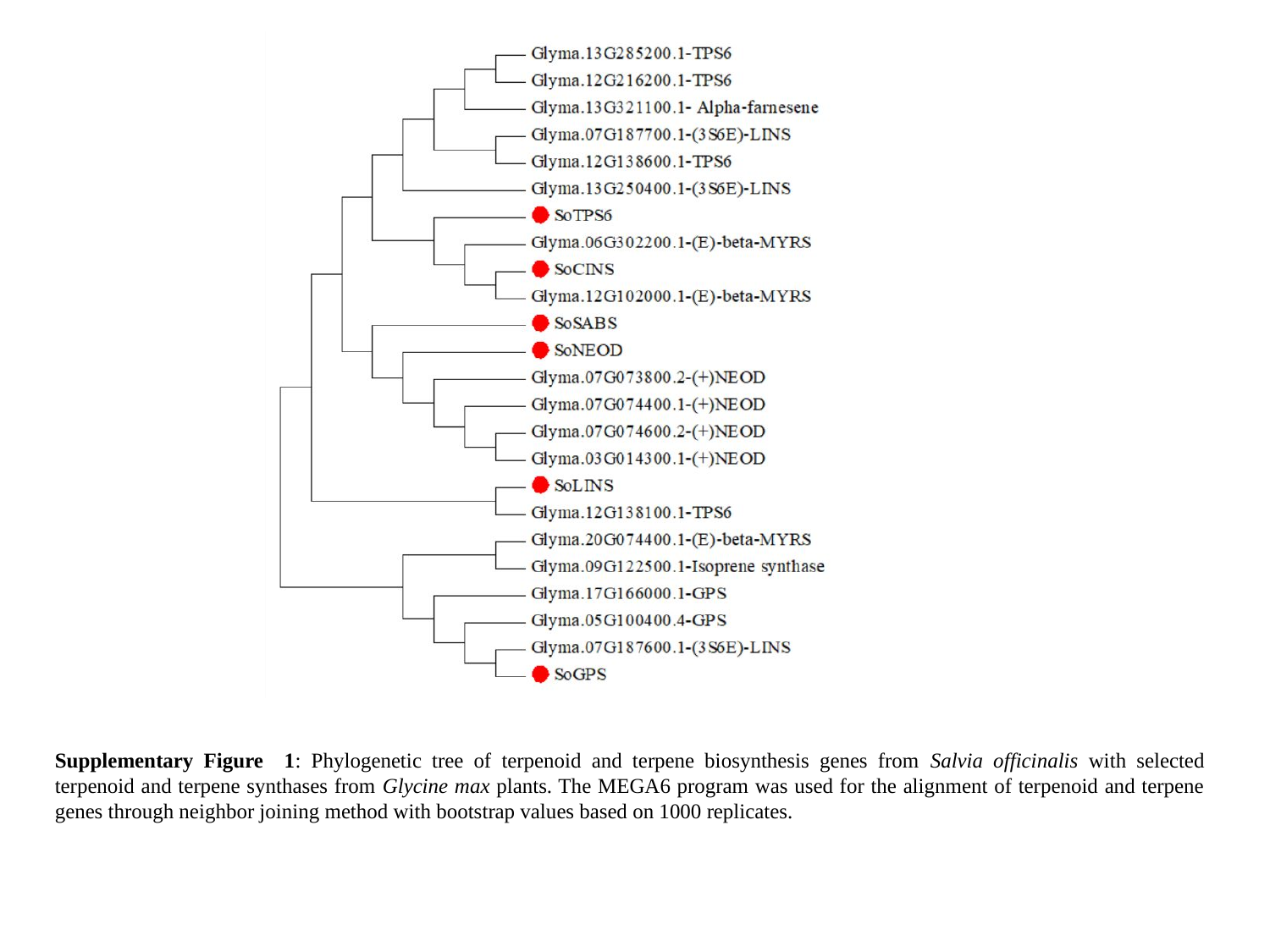

Supplementary Figure 1: Phylogenetic tree of terpenoid and terpene biosynthesis genes from Salvia officinalis with selected terpenoid and terpene synthases from Glycine max plants. The MEGA6 program was used for the alignment of terpenoid and terpene genes through neighbor joining method with bootstrap values based on 1000 replicates.

## Slide 2
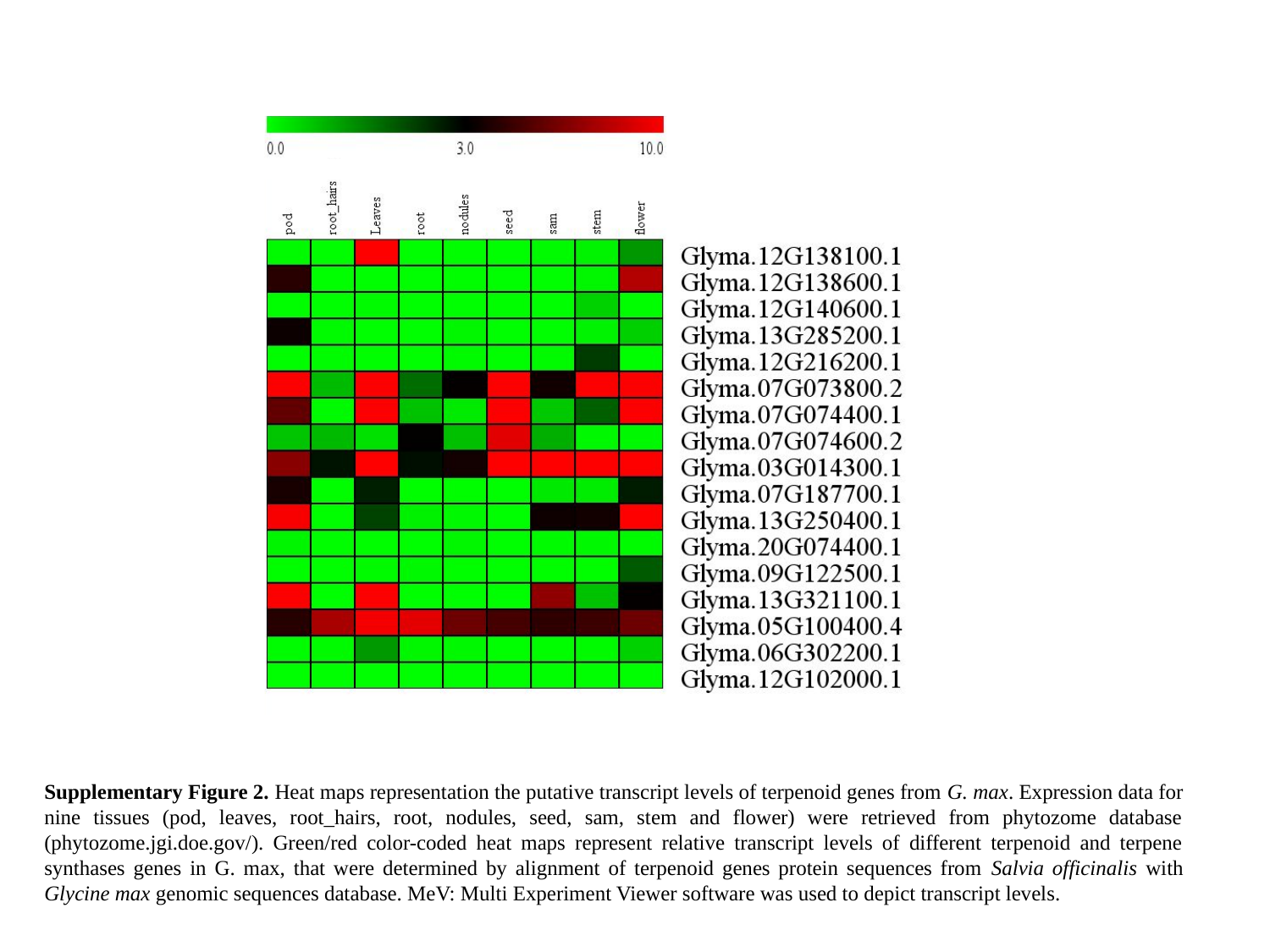

Supplementary Figure 2. Heat maps representation the putative transcript levels of terpenoid genes from G. max. Expression data for nine tissues (pod, leaves, root_hairs, root, nodules, seed, sam, stem and flower) were retrieved from phytozome database (phytozome.jgi.doe.gov/). Green/red color-coded heat maps represent relative transcript levels of different terpenoid and terpene synthases genes in G. max, that were determined by alignment of terpenoid genes protein sequences from Salvia officinalis with Glycine max genomic sequences database. MeV: Multi Experiment Viewer software was used to depict transcript levels.

## Slide 3
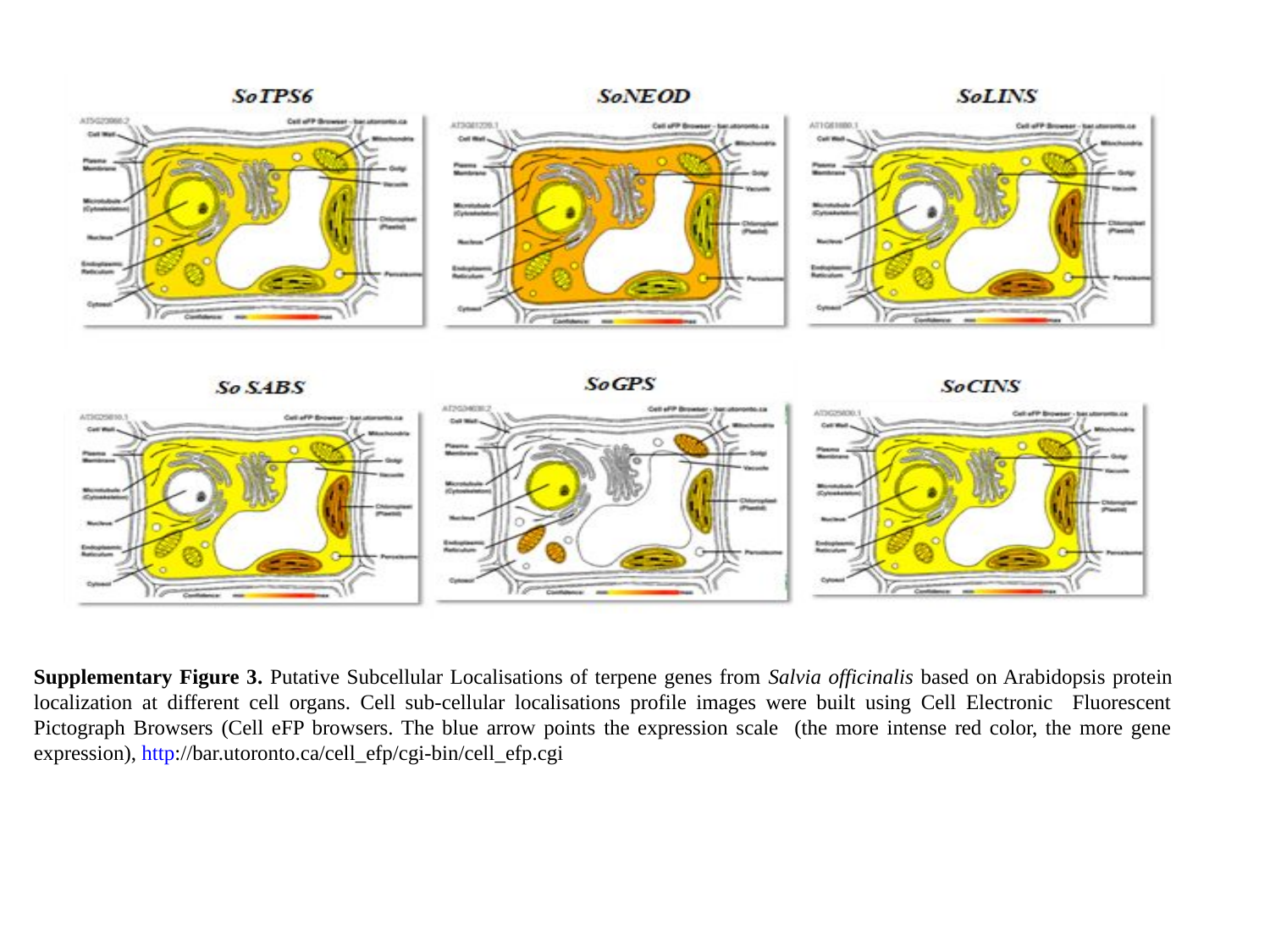

Supplementary Figure 3. Putative Subcellular Localisations of terpene genes from Salvia officinalis based on Arabidopsis protein localization at different cell organs. Cell sub-cellular localisations profile images were built using Cell Electronic Fluorescent Pictograph Browsers (Cell eFP browsers. The blue arrow points the expression scale (the more intense red color, the more gene expression), http://bar.utoronto.ca/cell_efp/cgi-bin/cell_efp.cgi
