# Supplementary material for: Overexpression of Terpenoid Biosynthesis Genes From Garden Sage (Salvia officinalis) Modulates Rhizobia Interaction and Nodulation in Soybean
Source: Front Plant Sci. 2021 Dec 23;12:783269. doi: 10.3389/fpls.2021.783269 (PMC8733304; doi:10.3389/fpls.2021.783269)
Supplement: Supplementary file 2 [file Table_1.doc]

| **Gene name** | **Short primer sequence** | **Long primer sequence** |
| --- | --- | --- |
| ***SoTPS6*** | 5ʹ- GATGAAGATGATTCAACCCCAA-3ʹ | 5ʹ- GGGGACAAGTTTGTACAAAAAAGCAGGCTTC GATGAAGATGATTCAACC-3ʹ |
|  | 5ʹ- CTAGCTAGAAAGCATGAAGGGG-3ʹ | 5ʹ- GGGGACCACTTTGTACAAGAAAGCTGGGTCTAGCTAGAAAGCATGAA-3ʹ |
| ***SoNEOD*** | 5ʹ- ATGGCAGATGCACTTGTCCAG-3ʹ | 5ʹ- GGGGACAAGTTTGTACAAAAAAGCAGGCTTCATGGCAGATGCACTTGTC-3ʹ |
|  | 5ʹ- TTACAAATCACACAATTATTAGGAGGAG-3ʹ | 5ʹ- GGGGACCACTTTGTACAAGAAAGCTGGGTCACAATTATTAGGAGGAG-3ʹ |
| ***SoLINS*** | 5ʹ- AGAGATATGTTGATGAAAATGGAGC-3ʹ | 5ʹ- GGGGACAAGTTTGTACAAAAAAGCAGGCTTC AGAGATATGTTGATGA-3ʹ |
|  | 5ʹ- CCTAGGAGTGATTTGGCGAAG-3ʹ | 5ʹ- GGGGACCACTTTGTACAAGAAAGCTGGGTCCTAGGAGTGATTTGG-3ʹ |
| ***SoSABS*** | 5ʹ- GAATTCTCAGACAACATGGTTTTG-3ʹ | 5ʹ- GGGGACAAGTTTGTACAAAAAAGCAGGCTTC GAATTCTCAGACAACATG-3ʹ |
|  | 5ʹ- GTTCATCTCCTTCCACGCCT-3ʹ | 5ʹ- GGGGACCACTTTGTACAAGAAAGCTGGGTGTTCATCTCCTTCCACGC-3ʹ |
| ***SoCINS*** | 5ʹ- ATGTCGAGTCTTATAATGCAAGTTGTG-3ʹ | 5ʹ- GGGGACAAGTTTGTACAAAAAAGCAGGCTTC ATGTCGAGTCTTATAATG-3ʹ |
|  | 5ʹ- TCATAGCGGTGGAACAGCAAG-3ʹ | 5ʹ- GGGGACCACTTTGTACAAGAAAGCTGGGTTCATAGCGGTGGAACAGC-3ʹ |
| ***SoGPS*** | 5ʹ- ATGATGTCGGTGAGAGGGCTC-3ʹ | 5ʹ- GGGGACAAGTTTGTACAAAAAAGCAGGCTTC ATGATGTCGGTGAGAGGG-3ʹ |
|  | 5ʹ- CTATTTTGTTCTGGTGATGACTATATGTGT-3ʹ | 5ʹ- GGGGACCACTTTGTACAAGAAAGCTGGGTCTATTTTGTTCTGGTGAT-3ʹ |

[**Supplementary Table S**](http://dnaresearch.oxfordjournals.org/lookup/suppl/doi:10.1093/dnares/dst051/-/DC1)**1**. List of *S. officinalis* genes and primer pairs used for full-length terpene synthases cDNAs clones.

[Supplementary Table S](http://dnaresearch.oxfordjournals.org/lookup/suppl/doi:10.1093/dnares/dst051/-/DC1)2. List of *Glycine max* and *S. officinalis* genes and primer pairs used for qRT-PCR.

| **Gene** | **Primer name** | **Primer sequence** | **PCR product (bp)** |
| --- | --- | --- | --- |
| ***GmACTIN*** | GmACTIN -F | 5ʹ- CTTCCCTCAGCACCTTCCAA -3ʹ | 140 |
|  | GmACTIN-R | 5ʹ- GGTCCAGCTTTCACACTCCAT -3ʹ |  |
| ***SoTPS6*** | SoTPS6-F | 5ʹ- TGAGGATACACTTCAAAGCCC-3ʹ | 158 |
|  | SoTPS6-R | 5ʹ- GTACATCTCAGCCATCCTTATCAT-3ʹ |  |
| ***SoNEOD*** | SoNEOD -F | 5ʹ- GTCAATGTCTCCTCCACTTTAG -3ʹ | 153 |
|  | SoNEOD -R | 5ʹ- CTCTTGCAGTTTACCCTCTTT-3ʹ |  |
| ***SoLINS*** | SoLINS -F | 5ʹ- AGAATTGGTGAAGGCAGAGG-3ʹ | 155 |
|  | SoLINS -R | 5ʹ- GTAGGATGTGGGTCTGATTGG-3ʹ |  |
| ***SoSABS*** | SoSABS -F | 5ʹ- CAACGCCAAAGTTTCGATATCC-3ʹ | 150 |
|  | SoSABS -R | 5ʹ- GCAAGCCTTAAAATCATTCCCG-3ʹ |  |
| ***SoGPS*** | SoGPS-F | 5ʹ- CTGGACAAACGGCAGAAG -3ʹ | 150 |
|  | SoGPS-R | 5ʹ- CAATCCCGTGGCGAATATC -3ʹ |  |
| ***SoCINS*** | SoCINS-F | 5ʹ- GGTGTTGCAGGAAGAAGTAG -3ʹ | 161 |
|  | SoCINS-R | 5ʹ- CTGTTGAGTACAGATCCCTTTC -3ʹ |  |

[Supplementary Table S](http://dnaresearch.oxfordjournals.org/lookup/suppl/doi:10.1093/dnares/dst051/-/DC1)3.List of *Glycine max* genes involved in nodules biosynthesis and signaling pathway and primer pairs used for qRT-PCR.

| **Gene name** | **Primer name** | **Primer sequence** | **PCR product (bp)** |
| --- | --- | --- | --- |
| *GmMAX1a* | *GmMAX1a-F* | 5ʹ- CCTCTCTAGTCCCCATTCAGTTT-3ʹ | 150 |
|  | *GmMAX1a-R* | 5ʹ- TGTGTTGGTTGATGAAATCTGATAC-3ʹ |  |
| *GmMAX1b* | *GmMAX1b-F* | 5ʹ- TACCGCAAATATTCTTCAGACC-3ʹ | 155 |
|  | *GmMAX1b-R* | 5ʹ- ACCAGATTCTATCCCTTTCACC-3ʹ |  |
| *GmMAX2* | *GmMAX2-F* | 5ʹ- TGTTTGCTCGCCAGGACTC-3ʹ | 160 |
|  | *GmMAX2-R* | 5ʹ- CACCATCACCCGGTTCATC-3ʹ |  |
| *GmMAX3* | *GmMAX3-F* | 5ʹ- CAGGCTACTCACCGTGTCTT-3ʹ | 152 |
|  | *GmMAX3-R* | 5ʹ- GGTATCCGTTACAGCCCAAT-3ʹ |  |
| *GmMAX4a* | *GmMAX4a-F* | 5ʹ- CTTTCACCACCTTGCCTTCA-3ʹ | 148 |
|  | *GmMAX4a-R* | 5ʹ- TGGTTTCTTTCCGTTCCTCC-3ʹ |  |
| *GmMAX4b* | *GmMAX4b-F* | 5ʹ- GAAATAAAGGCATCTACAAAGGGAA-3ʹ | 150 |
|  | *GmMAX4b-R* | 5ʹ- ATGGTGGTGGTGGTGGCAGT-3ʹ |  |
| *GmNINa* | *GmNINa-F* | 5ʹ- TAACATGCGATGCTGATCTTG-3ʹ | 150 |
|  | *GmNINa-R* | 5ʹ- TGATTTAGAGGCGAAGCTTGA-3ʹ |  |
| *GmNINb* | *GmNINb-F-* | 5ʹ- CATGGAGTCGACGCAAATAA-3ʹ | 160 |
|  | *GmNINb-R* | 5ʹ- TCAAGTACCCAACAGCAATC-3ʹ |  |
| *GmNFR5* | *GmNFR5-F* | 5ʹ- TTCCCTTTCTTCCTCTCCAC-3ʹ | 149 |
|  | *GmNFR5-R* | 5ʹ- GCATGAAAAGTTTGTTCTATTGTC-3ʹ |  |
| *GmNSP1a* | *GmNSP1a-F* | 5ʹ- CAACACTTATCTTCTTCTCCAACT-3ʹ | 160 |
|  | *GmNSP1a-R* | 5ʹ- GGAAGCATTTGCTATGTTGTTAGG-3ʹ |  |
| *GmNSP1b* | *GmNSP1b-F* | 5ʹ- ATCCTCGTCTTCTTCCAAATAC-3ʹ | 155 |
|  | *GmNSP1b-R* | 5ʹ- GGGAGAAGGAGTAGGAGTAAT-3ʹ |  |
| *GmNSP2a* | *GmNSP2a-F* | 5ʹ- GAACTTACCGCACCTTAGTT-3ʹ | 155 |
|  | *GmNSP2a-R* | 5ʹ- GATGCAGCGACTCCATAAA-3ʹ |  |
| *GmNSP2b* | *GmNSP2b-F* | 5ʹ- AATCATTGCCAAGCGAAGCT-3ʹ | 149 |
|  | *GmNSP2b-R* | 5ʹ- AGTCCAAAGCGAGGCAGAGA-3ʹ |  |
| *GmNSP2b* | *GmDMI2a-F* | 5ʹ- GTCCTCAGTGGCCTTGACATT-3ʹ | 160 |
|  | *GmDMI2a-R* | 5ʹ- ACACCCTTTTGCCTGCTTTG-3ʹ |  |
| *GmDMI2b* | *GmDMI2b-F* | 5ʹ- ATTCACGAGCACACTGTGCCT-3ʹ | 158 |
|  | *GmDMI2b-R* | 5ʹ- CCAAAATCTGCAACCTTTCC-3ʹ |  |
| *GmDMI3α* | *GmDMI3α-F* | 5ʹ- AGTGTTTGGAGCACCGCAATC-3ʹ | 147 |
|  | *GmDMI3α-R* | 5ʹ- TCAAACAAGTCAAATATACGTGGTG-3ʹ |  |
| *GmDMI3b* | *GmDMI3b-F* | 5ʹ- TTGTCCATAGGGAGGGTAAT-3ʹ | 159 |
|  | *GmDMI3b-R* | 5ʹ- ATGAGTAGAAGGGTAGCTAGAG-3ʹ |  |
